# Supplementary material for: Increasing central and northern European summer heatwave intensity due to forced changes in internal variability
Source: Nat Commun. 2025 Oct 30;16:9485. doi: 10.1038/s41467-025-65392-w (PMC12575768; doi:10.1038/s41467-025-65392-w)
Supplement: Supplementary file 1 — Supplementary Information [file 41467_2025_65392_MOESM1_ESM.pdf]

Supplementary Information

for

Increasing central and northern European summer heatwave intensity due to forced changes in internal variability

Goratz Beobide-Arsuaga[1*](goratz.beobide.arsuaga@uni-hamburg.de), Laura Suarez-Gutierrez[2,3], Armineh Barkhordarian[1], Dirk Olonscheck[4], Johanna Baehr[1]

[1]Institute of Oceanography, Center for Earth System Research and Sustainability (CEN), Universität Hamburg, Hamburg, Germany

[2]Institute for Atmospheric and Climate Science, ETH Zurich, Zurich, Switzerland

[3]Laboratoire des Sciences du Climat et de l'Environnement, Institut Pierre-Simon Laplace, Paris, France

[4]Max Planck Institute for Meteorology, Hamburg, Germany

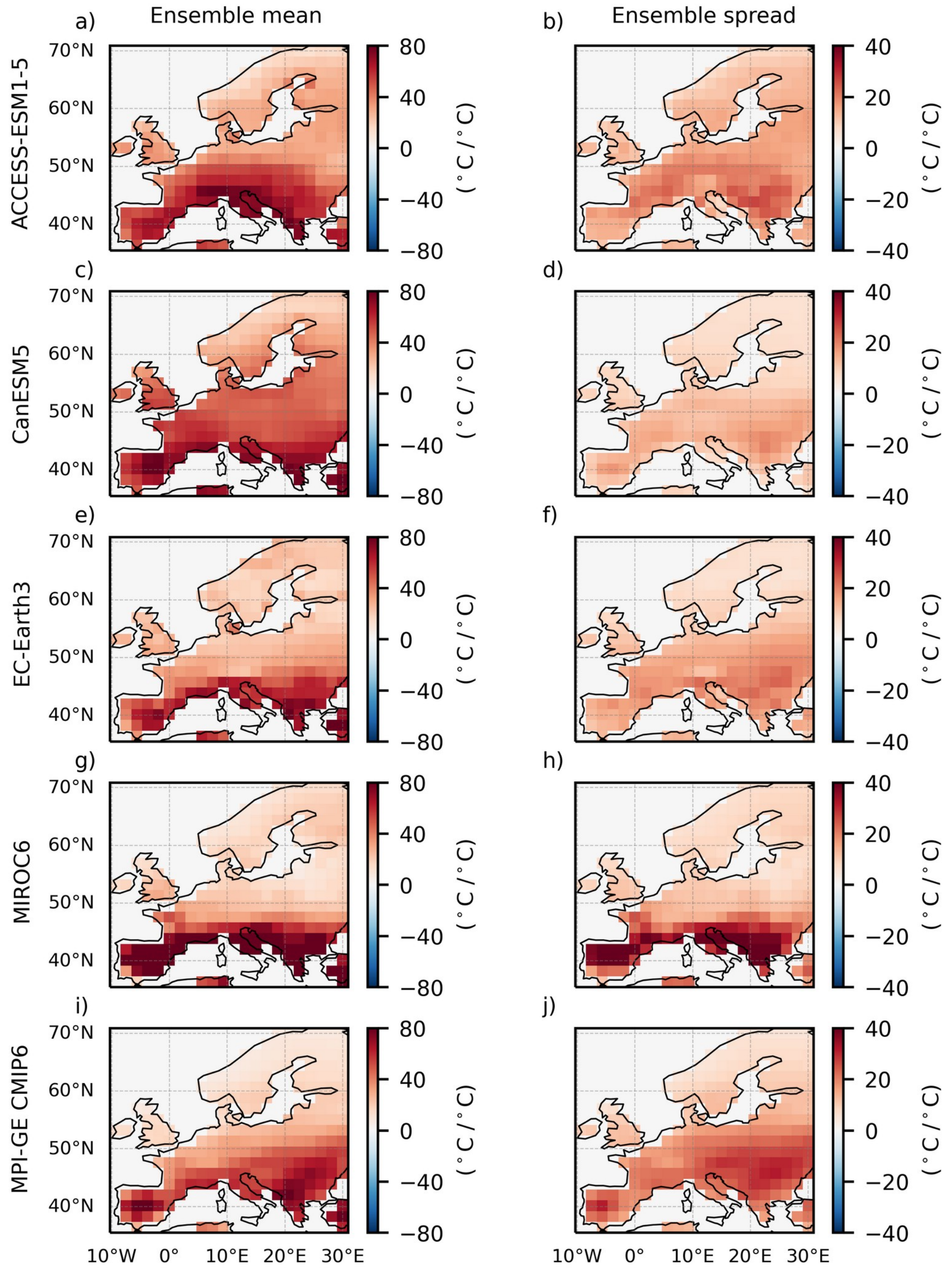

Supplementary Figure 1: ***Spatial distribution of changes in European summer heatwave (EuSHW) intensity under global warming levels.*** *Regression coefficients for the period 2014-2100 and* for shared socioeconomic pathway (SSP) *2-4.5 and SSP5-8.5 scenarios between global mean temperature anomalies relative to 1985-2014 and; a) the forced signal (i.e., ensemble mean) of non-detrended EuSHW cumulative heat for ACCESS-ESM-1.5; b) the range (i.e., ensemble spread computed as ensemble standard deviation) of non-detrended EuSHW cumulative heat due to internal variability for ACCESS-ESM-1.5; c,d) same as a,b) but for CanESM5; e,f) same as a,b) but for EC-Earth3; g,h) same as a,b) but for MIROC6; i,j) same as a,b) but for MPI-GE CMIP6. The non-dashed regions show significant changes at the 95% confidence level.*

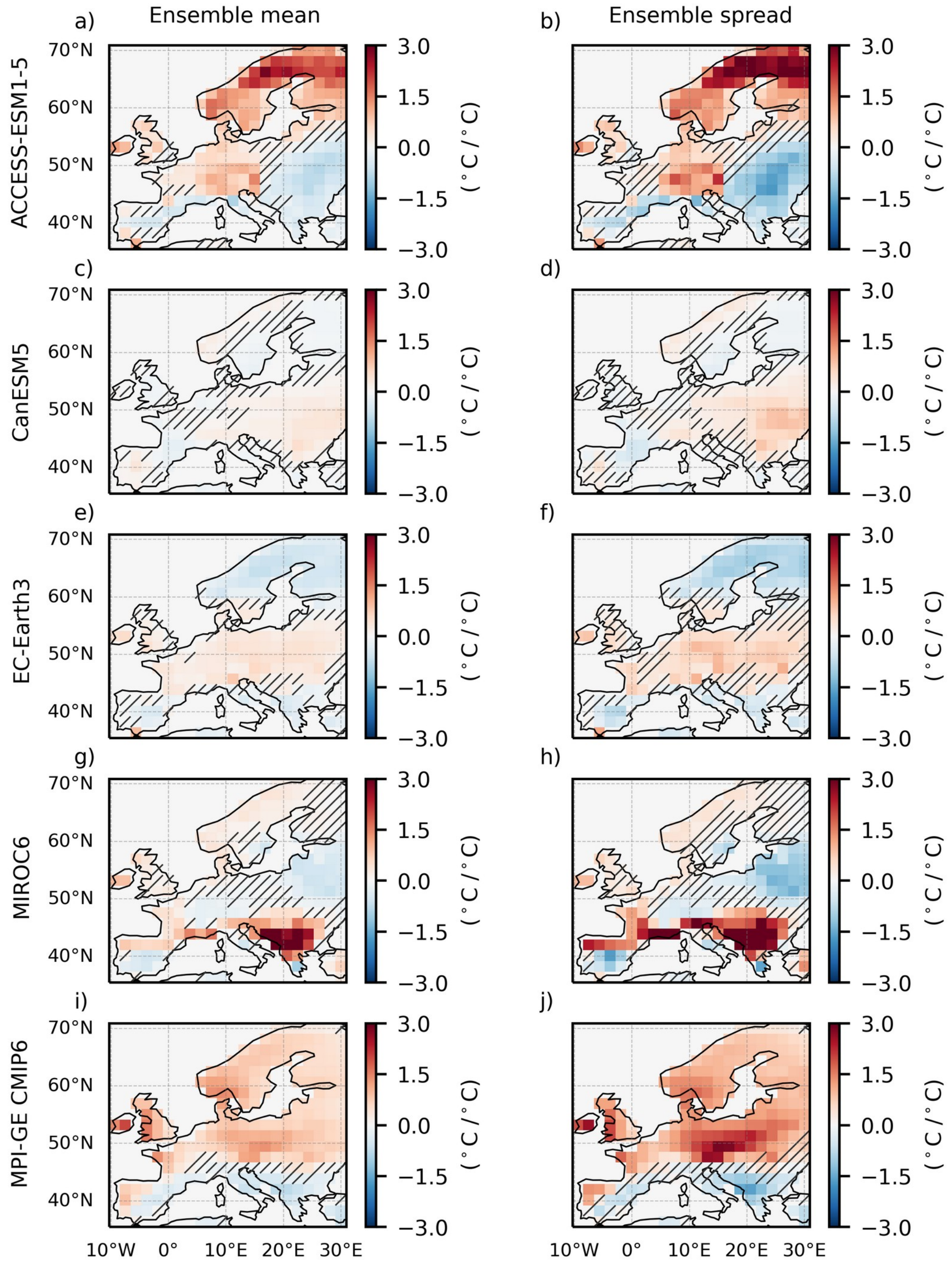

Supplementary Figure 2: ***Spatial distribution of changes in European summer heatwave (EuSHW) intensity due to forced changes in internal variability under global warming levels.*** *Regression coefficients for the period 2014- 2100 and* for shared socioeconomic pathway (SSP) *2-4.5 and SSP5-8.5 scenarios between global mean temperature anomalies relative to 1985-2014 and; a) the forced signal (i.e., ensemble mean) of detrended EuSHW cumulative heat for ACCESS-ESM-1.5; b) the range (i.e., ensemble spread computed as ensemble standard deviation) of detrended EuSHW cumulative heat due to internal variability for ACCESS-ESM-1.5; c,d) same as a,b) but for CanESM5; e,f) same as a,b) but for EC-Earth3; g,h) same as a,b) but for MIROC6; i,j) same as a,b) but for MPI-GE CMIP6. The non-dashed regions show significant changes at the 95% confidence level.*

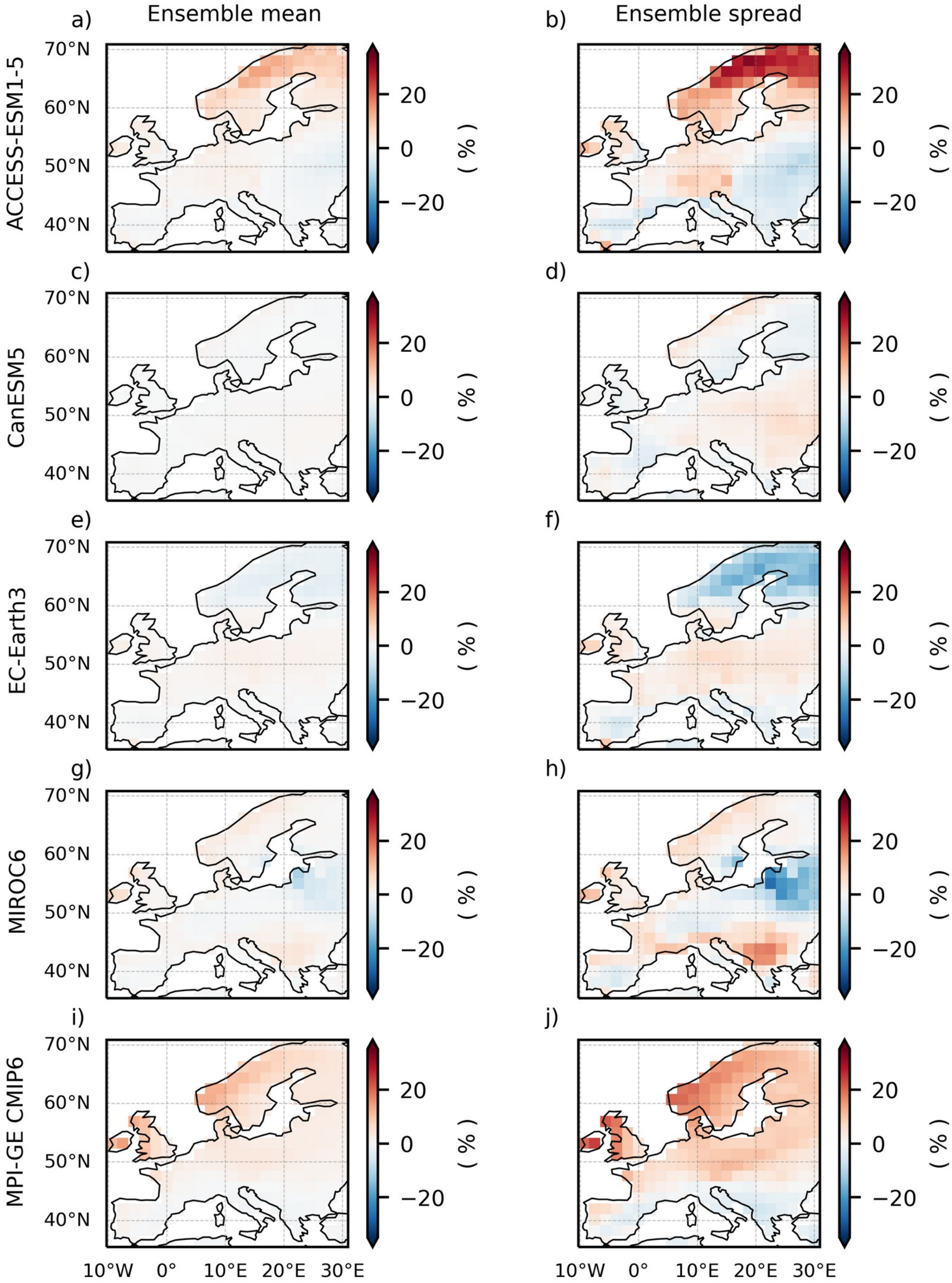

Supplementary Figure 3: ***The percentage contribution of forced changes in internal variability.*** *The percentage ratio of detrended to non-detrended EuSHW intensity changes with global mean temperature anomalies (relative to 1985-2014) during the period 2014- 2100 under* shared socioeconomic pathway (SSP) *2-4.5 and SSP5-8.5 scenarios. The percentage ratios are shown for; a) the forced signal (i.e., ensemble mean) for ACCESS-ESM-1.5; b) the range (i.e., ensemble spread computed as ensemble standard deviation) for ACCESS-ESM-1.5; c,d) same as a,b) but for CanESM5; e,f) same as a,b) but for EC-Earth3; g,h) same as a,b) but for MIROC6; i,j) same as a,b) but for MPI-GE CMIP6.*

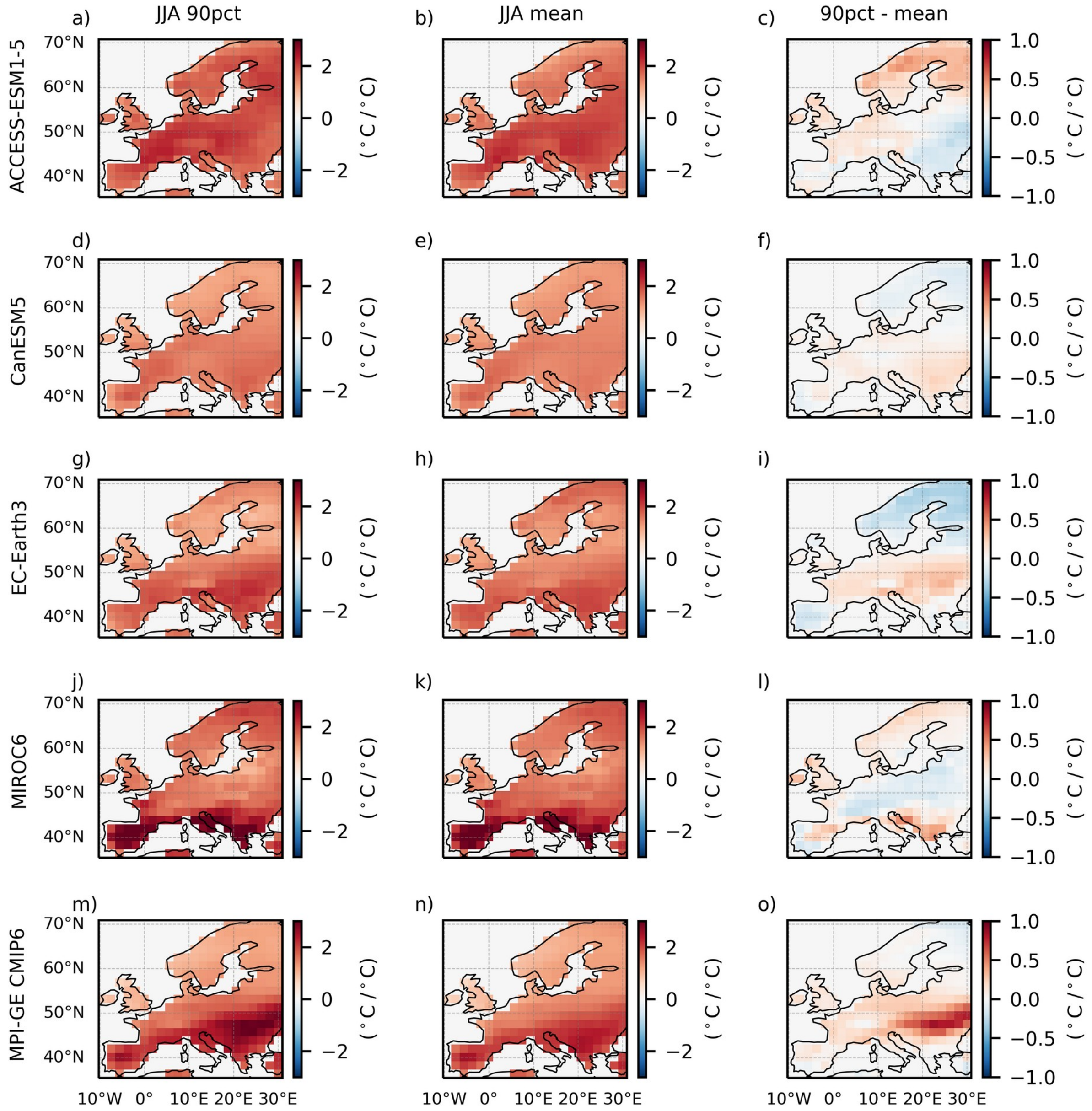

Supplementary Figure 4: ***Spatial distribution of the forced signal of summer daily maximum 2m air temperatures (T2max) under global warming levels.*** *Regression coefficients between global mean temperature anomalies relative to 1985-2014 and; a) the forced signal (i.e., ensemble mean) of 90th percentile summer (June, July, August) T2max for ACCESS-ESM-1.5; b) the forced signal (i.e., ensemble mean) of mean summer (June, July, August) T2max for ACCESS-ESM-1.5; c) the difference between a) and b); d-f) same as a-c) but for CanESM5; g-i) same as a-c) but for EC-Earth3; j-l) same as a-c) but for MIROC6; m-o) same as a-c) but for MPI-GE CMIP6.*

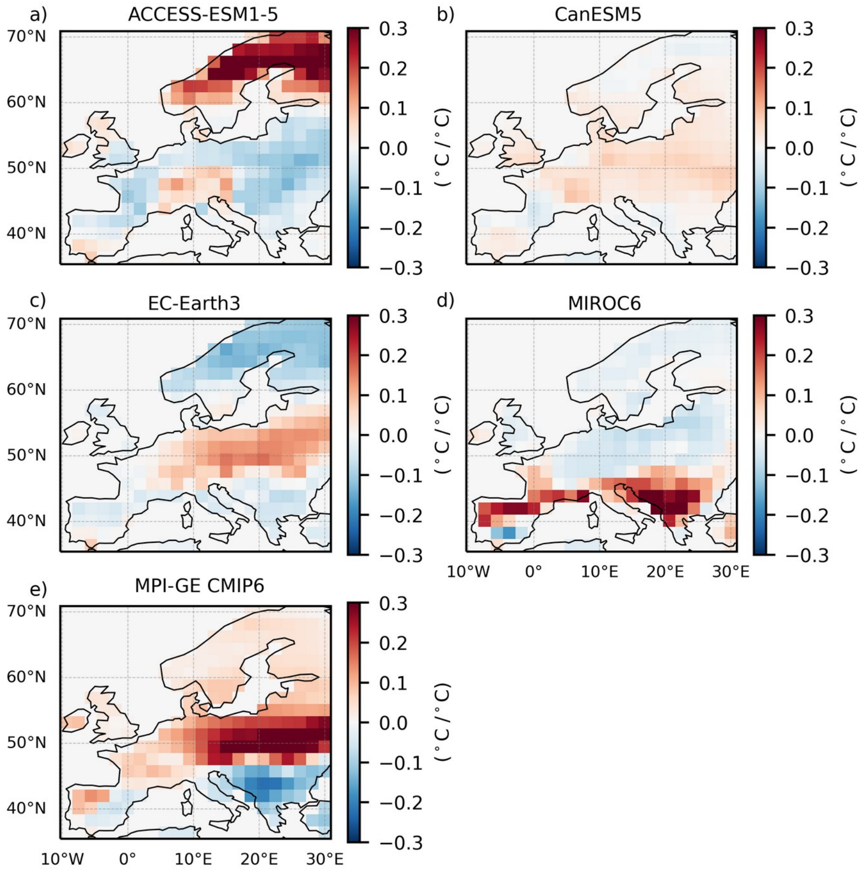

Supplementary Figure 5: ***Spatial distribution of extreme summer temperature variability changes under global warming levels.*** *Regression coefficients between global mean temperature anomalies relative to 1985-2014 and the range (i.e. ensemble spread computed as ensemble standard deviation) of 90th percentile summer (June, July, August) daily maximum 2m air temperatures for; a) ACCESS-ESM-1.5; b) CanESM5; c) EC-Earth3; d) MIROC6; e) MPI-GE CMIP6.*

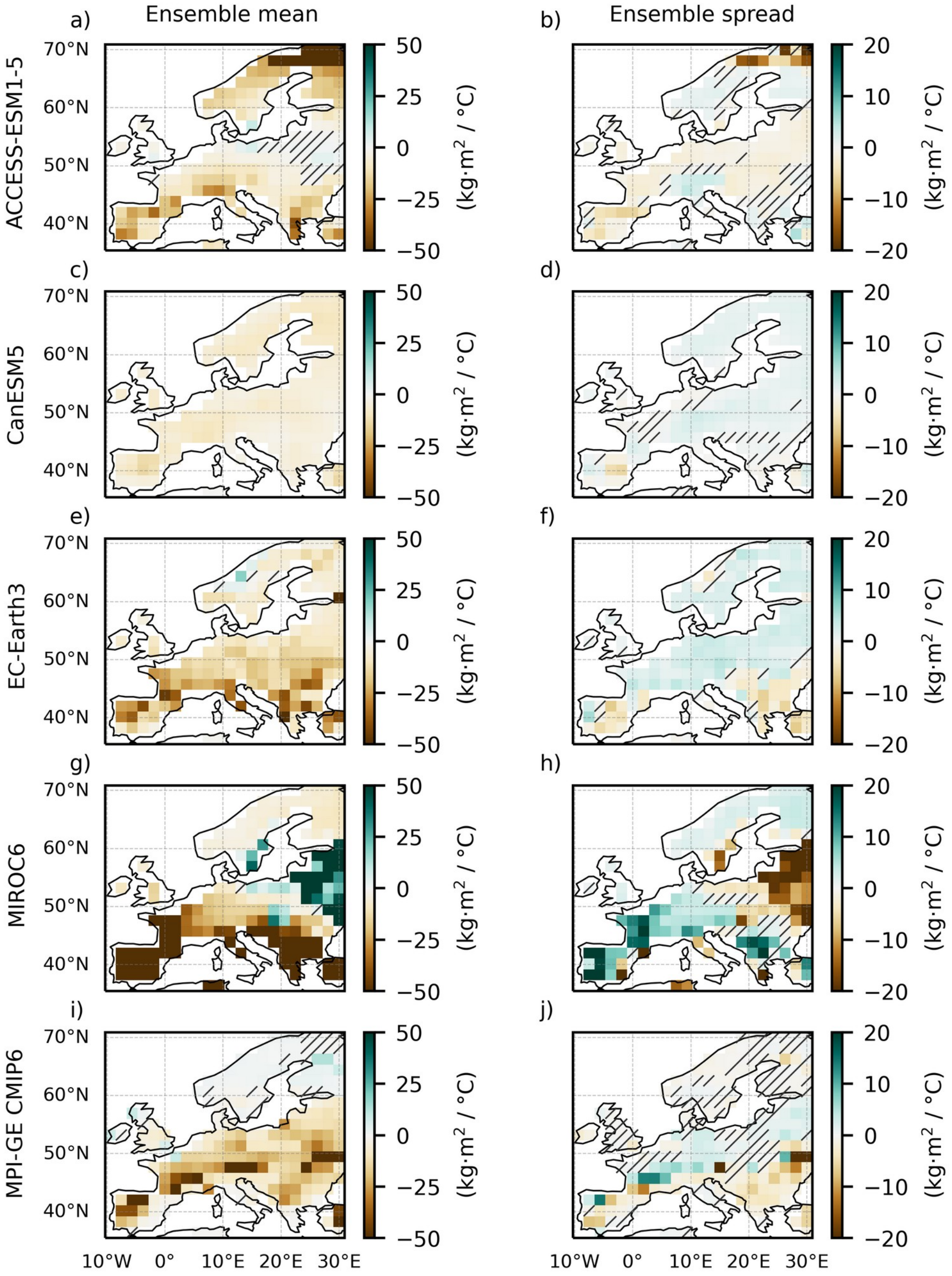

Supplementary Figure 6: ***Spatial distribution of changes in soil moisture under global warming levels.*** *Regression coefficients for the period 2014- 2100 and* for shared socioeconomic pathway (SSP) *2-4.5 and SSP5-8.5 scenarios between global mean temperature anomalies relative to 1985-2014 and; a) the forced signal (i.e., ensemble mean) of summer (June, July, August) mean soil moisture; b) the range (i.e., ensemble standard deviation) of summer mean soil moisture for ACCESS-ESM-1.5; c,d) same as a,b) but for CanESM5; e,f) same as a,b) but for EC-Earth3; g,h) same as a,b) but for MIROC6; i,j) same as a,b) but for MPI-GE CMIP6. The non-dashed regions show significant changes at the 95% confidence level.*
